# Couverture, cartographie et barrières à la vaccination complète pour l’âge chez les enfants de moins de cinq ans en 2021 : cas des localités d'Adjara-Hounvè et Ahouicodji au sud du Bénin

**DOI:** 10.48327/mtsi.v4i1.2024.352

**Published:** 2024-01-25

**Authors:** Barikissou Georgia DAMIEN, Wenceslas VL AVON OU, Marlène DAHOUN, Landry KAUCLEY, Badirou AGUEMON

**Affiliations:** 1 Département Population et santé, Centre de formation et de recherche en matière de population, Université d'Abomey-Calavi, Cotonou, Bénin; 2 Ministère de la Santé, Agence nationale des soins de santé primaires (ANSSP), Direction de la vaccination et de la logistique, Cotonou, Bénin; 3 Département de santé publique, Faculté des sciences de la santé, Université d'Abomey-Calavi, Cotonou, Bénin

**Keywords:** Vaccination complète pour l’âge, Distribution spatiale, Facteurs associés, Enfants de 0 à 5 ans, Adjara-Hounvè, Ahouicodji, Pahou, Ouidah, Bénin, Afrique subsaharienne, Full immunization for age, Mapping, Associated factors, Children aged 0-5 years, Adjara-Hounvè, Ahouicodji, Pahou, Ouidah, Benin, Sub-Saharan Africa

## Abstract

**Introduction:**

La vaccination des enfants est primordiale afin de réduire le taux de morbidité et de mortalité infantile. Par ailleurs, l'inaccessibilité aux soins, surtout pendant la période critique de la pandémie Covid-19, a fortement réduit les taux de couverture vaccinale. Lobjectif de notre étude était détudier la couverture et les facteurs associés à la vaccination complète pour l’âge chez les enfants de moins de 5 ans dans l'arrondissement de Pahou dans la commune de Ouidah au Bénin en 2021.

**Méthodologie:**

Une étude transversale descriptive et analytique a été réalisée dans les villages d'Adjara-Hounvè et Ahouicodji dans l'arrondissement de Pahou avec un recrutement exhaustif des ménages. L'enquête a porté sur les enfants de moins de 5 ans pour lesquels un carnet de vaccination était présenté. Le questionnaire a été numérisé. L'analyse descriptive et la recherche de facteurs associés ont été réalisées à l'aide d'un modèle de régression logistique grâce au logiciel Stata/SE 14 et la cartographie à l'aide du logiciel ArcGIS 10.8.

**Résultats:**

Sur les 414 mères enquêtées, les informations ont été recueillies chez les 238 enfants de 0 à 5 ans (57,49 %) possédant un carnet de vaccination. Sur les 238 enfants, 20,6 % avaient une vaccination complète pour leur âge. Le niveau d'instruction « primaire » *versus* « aucun » (ORa = 3,32; IC95% 1,07-10,25), la profession « personnel de santé » *versus* « ménagère » (ORa = 21,18; IC95% 3,07-145,94), la connaissance des maladies du PEV par les mères (ORa = 2,20; IC95% 1,03-4,68) et l’âge des enfants 0-2 mois *versus* ≥ 16 mois (ORa = 8,53; IC95% 2,52-28,85) et 9-15 mois *versus* ≥ 16 mois (ORa = 2,99; IC95% 1,24-7,23) ont augmenté le statut vaccinal complet pour l’âge. Une tendance à l'homogénéité du comportement lié à la couverture vaccinale complète pour l’âge chez les enfants de moins de 5 ans a été mise en évidence à la cartographie.

**Conclusion:**

La couverture vaccinale complète pour l’âge chez les enfants de moins de 5 ans est très faible, avec une tendance d'homogénéité spatiale dans le comportement de recours à la vaccination par la communauté. La couverture vaccinale complète pour l’âge est un indicateur innovant pouvant contribuer à atteindre les objectifs vaccinaux pour chaque âge.

## Abstract (en)

**Background:**

Vaccination is a protective measure against infectious diseases and remains one of the best investments in public health. Some African countries are still struggling to reach the required child immunization coverage. Several factors are responsible for limiting immunization coverage. Most of the factors considered to limit immunization coverage are related to the health system. In addition, inaccessibility to care, especially during the critical period of the Covid-19 pandemic, greatly reduced vaccination coverage rates. In Benin, several vaccines are included in the Expanded Programme on Immunization or are administered as part of routine immunization. However, cases of non-compliance with the vaccine and persistent flaccid paralysis are still recorded in the commune of Ouidah in southern Benin. The aim of this study was to investigate the coverage and factors associated with full immunization for age in children aged 0-5 years.

**Methods:**

A cross-sectional survey was conducted from August to October 2021 in two villages (Adjara-Hounvè and Ahouicodji) in southern Benin. All the households were included. The survey regarded children under 5 for whom a vaccination record was presented. A couple child/mother was recruited after informed consent of the mother and her child. An univariate analysis followed by a multivariate analysis was performed by using a logistic regression model to identify the variables that influence vaccine completeness. Spatial description of vaccine completeness was performed using the kriging method using ArcGIS 10.8 mapping software. Results. Of the 414 mothers surveyed, 57.49% had an immunization card, from which information was collected. Of the 238 children recruited, 141 were in Adjara-Hounvè and 97 in Ahouicodji. Of the 238 children with an immunization card, 20.6% were fully immunized for their age. All children received Baccille Calmette Guérin vaccine at birth. Since poliomyelitis, pentavalent, pneumococcal conjugate, and rotavirus are three-dose vaccines, the percentage of children who received these vaccines decreased as the number of doses increased: 96.6%, 88.2%, 78.1% and 72.3% for the four doses of polio respectively. According to 53.4% of the respondents the reception at the vaccination site was poor, and according to 70.3% of them waiting time for vaccination sessions was long. Several reasons justified the absence of complete vaccination for the age of the children: vaccination site too far from the place of residence (59.54%), lack of financial means (29.78%) and the mother's ignorance (12.76%). Education level “primary” *vs* “none” (ORa = 3.32; CI95% 1.07-10.25), occupation “health staff” *vs* “housewife” (ORa = 21.18; CI95% 3.07-145.94), mothers’ knowledge of Expanded Programme on Immunization diseases (ORa = 2, 20; CI95% 1.03-4.68) and children's age 0-2 months vs ≥ 16 months (ORa = 8.53; CI95% 2.52-28.85) and 9-15 months vs ≥ 16 months (ORa = 2.99; CI95% 1.24-7.23) increased complete immunization status for age. The homogeneity of behaviour related to age-complete immunization coverage in children under 5 years was evident at mapping.

**Conclusion:**

Age-complete immunization coverage in children under 5 years of age is very low, with a spatial homogeneity in community immunization uptake behaviour. Age-complete immunization coverage is an innovative indicator that can contribute to achieving age-specific immunization targets.

## Introduction

La vaccination est une mesure de prévention efficace contre les maladies infectieuses [4] et représente l'un des meilleurs investissements en matière de santé publique [14]. La vaccination des enfants de moins de 5 ans a pour but de réduire la morbidité et la mortalité infantile à moindre coût [27]. Certains pays africains ont encore du mal à atteindre la couverture vaccinale infantile requise [4, 38], qui est selon l'Organisation mondiale de la Santé de 90 % au niveau national et de 80 % dans chaque district ou unité administrative équivalente, pour tous les vaccins inclus dans les programmes nationaux, sauf recommandation contraire [28].

À l’échelle mondiale en 2020, la couverture vaccinale a baissé, passant de 86 % en 2019 à 83 % en 2020 correspondant à 23 millions d'enfants de moins d'un an qui n'ont pas reçu leurs premières doses de vaccin [29]. Par ailleurs, des millions d'enfants n'ont pas reçu les vaccins de base dans le cadre du Programme élargi de vaccination (PEV) [31] mis en œuvre au Bénin par la Direction de la vaccination et de la logistique (DVL) sous l'autorité de l'Agence nationale des soins de santé primaires (ANSSP). Cette même tendance a été observée en 2021 et en 2022 [29, 30, 31] probablement due à la pandémie de Covid-19 et aux perturbations en lien qui ont mis à rude épreuve les systèmes de santé. Plusieurs facteurs sont responsables de la diminution de la couverture vaccinale. La plupart de ces facteurs sont liés au système de santé. Il s'agit du manque de personnel [5], et de l'incapacité pour les pays d'Afrique subsaharienne d'atteindre les populations d'enfants les plus vulnérables dans les communautés rurales et isolées [6]. Par ailleurs, l'inaccessibilité aux soins, surtout pendant la période critique de la pandémie de Covid-19, a fortement réduit les couvertures vaccinales. La deuxième barrière à une bonne couverture vaccinale la plus évoquée ces 20 dernières années est l'acceptation des vaccins par les populations. L'hésitation vaccinale serait liée en partie au type de vaccins, aux personnes, groupes sociaux ou géographiques, et au contexte [6, 16].

Au Bénin, selon les enquêtes démographiques de santé (EDS) [19], qui se fondent sur les carnets de vaccination ou les déclarations des mères, 57 % des enfants de 12-23 mois ont reçu tous les vaccins de base (BCG et vaccins contre la poliomyélite, la diphtérie, le tétanos, la coqueluche, l’*Haemophilus influenzae* type B, la rougeole et la fièvre jaune) et 51 % ont reçu tous les vaccins appropriés pour leur groupe d’âge. À l'opposé, 11 % des enfants n'ont reçu aucun vaccin [21]. Dans le département de l'Atlantique où se situe la zone d’étude, les enfants ayant reçu tous les vaccins appropriés pour leur groupe d’âges étaient de 58,9 % [21]. Plus spécifiquement, 68,4 % des enfants ont reçu 4 doses de vaccin contre la poliomyélite. En 2021, un plan de riposte vaccinale contre l’épidémie de poliomyélite a été déployé dans 7 départements et 39 communes, dont la commune de Ouidah. Il s'agit d'une campagne de masse. Cependant, les cas de non-compliance vaccinale et de persistances de paralysies flasques demeurent enregistrés dans la commune de Ouidah [23]. Par ailleurs, très peu d’études ont abordé la couverture vaccinale en termes de statut vaccinal complet pour l’âge. Les plus récentes études réalisées au Bénin concernent la revue complète du PEV couplée à une enquête nationale de couverture vaccinale en 2014 et les EDS de 2012 et de 2018-2019. Elles ont montré que la pauvreté, le département de résidence, le niveau d'instruction des parents, la distance parcourue par les mères pour se rendre au centre de santé, le lieu d'accouchement, l'assistance à l'accouchement, les visites prénatales, l'appartenance ethnique de la mère peuvent expliquer cette réticence, mais aussi d'autres facteurs tels que le manque de tact des vaccinateurs, les expériences antérieures des parents et les fausses rumeurs sur la vaccination associées à la couverture vaccinale chez l'enfant de 12 à 23 mois [8, 12, 13, 21]. Cette étude avait pour objectif d’étudier la couverture vaccinale et les facteurs associés à la vaccination complète pour l’âge chez les enfants de moins de 5 ans. Elle avait également pour but de contribuer à la mise en place de nouvelles approches de collecte, d'analyse et d'interprétation des données sur la vaccination en vue d'améliorer la couverture vaccinale au Bénin.

## Méthodologie

### Cadre d’étude

Pahou est un arrondissement de la commune de Ouidah localisé dans le département de l'Atlantique au sud du Bénin [20], L'arrondissement est composé de 9 villages et l’étude a été conduite dans les villages d'Adjara-Hounvè et Ahouicodji. En 2021, la population de l'arrondissement de Pahou était estimée à 19 800 habitants [20, 22]. Les effectifs d'enfants de moins de 5 ans des villages d'Adjara-Hounvè et Ahouicodji étaient respectivement de 596 et de 530. L'arrondissement de Pahou comprend 4 centres de santé publics : Kpovié, Acadjamè, Houndjava et Pahou-centre. Les relais communautaires (RC) sont chargés de mener des activités de sensibilisation, d'information, d’éducation et de communication dans la communauté. Trois RC étaient affectés dans chaque village de l’étude.

### Méthodes d’étude

Une étude transversale descriptive à visée analytique a été conduite d'août à octobre 2021. Deux cibles ont été considérées. La cible primaire était constituée par les enfants de moins de 5 ans et leurs critères d'inclusion étaient : i) résider dans le milieu d’étude depuis la naissance; ii) avoir son carnet de vaccination disponible; iii) être présent durant la période de l'enquête. La cible secondaire représentée par les mères des enfants sélectionnés pour l’étude avait pour critères d'inclusion : i) être la mère d'un enfant de 0 à 5 ans; ii) résider dans le milieu d’étude depuis la naissance de l'enfant; iii) être disponible durant la période de l'enquête; iv) avoir donné son consentement éclairé pour elle-même et pour son enfant.

Deux villages distants de 11,77 km ont été choisis dans l'arrondissement de Pahou, l'un à l'extrême nord (Adjara-Hounvè) et le second à l'extrême sud (Ahouicodji) afin d'observer la complétude vaccinale dans deux espaces géographiques non contigus. Dans une concession comportant plusieurs ménages ayant des enfants de 0 à 5 ans, tous les ménages ont été inclus. Dans les ménages ayant plus d'un enfant de moins de 5 ans, un seul enfant a été sélectionné de façon aléatoire.

La taille de l’échantillon de l’étude était de 262 et déterminée à partir de la formule de Schwartz : avec n la taille minimale de l’échantillon, £_a_ égal à 1,96 pour un niveau de confiance égal à 95 %, p la proportion estimée de la population qui a une complétude vaccinale au Bénin (57 %) [2] et i la marge d'erreur (fixée à 6 %). La variable dépendante était la complétude vaccinale pour l’âge chez les enfants de moins de 5 ans. Les variables indépendantes étaient regroupées en caractéristiques liées au ménage (indice de richesse, localisation géographique et distance par rapport au centre de santé/relais communautaire et nombre d'enfants); en caractéristiques sociodémographiques des enfants (âge et sexe); en caractéristiques sociodémographiques des mères (âge, niveau d'instruction et activité génératrice de revenus); en perception des mères (sur la complétude vaccinale des enfants, sur l'accueil, sur la distance, sur l'importance de la vaccination; en niveau de connaissance sur la vaccination (connaissance des maladies du PEV, connaissance du lieu de vaccination et information sur la vaccination) et la disponibilité des carnets de vaccination. En prélude à l'enquête, un prétest a été réalisé. Un entretien face à face a été effectué avec la cible secondaire de l'enquête à l'aide d'un questionnaire numérisé. Les coordonnées géographiques ont été prises à l'aide d'un GPS Garmin avec une précision de 3 m. Sur le plan descriptif, l'analyse a été faite aux moyens de logiciels spécialisés. Stata/SE 14.0 a permis d'analyser les données. Le taux de complétude vaccinale (TCV) a été déterminé en se servant du calendrier vaccinal du PEV pour l’âge de chaque enfant grâce à la formule : où TCV est le taux de couverture vaccinale complète, NECV le nombre d'enfants de 0 à 5 ans ayant une vaccination complète et NTE le nombre total d'enfants de 0 à 5 ans.

Pour identifier les variables qui influencent la complétude vaccinale, une analyse univariée suivie d'une analyse multivariée a été faite grâce à un modèle de régression logistique. Le logiciel Stata/SE 14.0 a été utilisé. Les associations ont été considérées comme statistiquement significatives au seuil critique de 5 %. La force de l'association a été mesurée par l'odds ratio avec son intervalle de confiance de 5 %. Sur le plan spatial, la dynamique de la complétude vaccinale a été réalisée grâce à la méthode de krigeage [11] en utilisant le logiciel de cartographie ArcGIS 10.8. La variable dépendante était la vaccination complète pour l’âge. Les enfants de moins de 5 ans complètement vaccinés pour leur âge ont été codés 1 et ceux incomplètement vaccinés pour leur âge codés 0.

Les variables indépendantes étaient relatives aux caractéristiques du ménage, aux caractéristiques sociodémographiques de l'enfant et de la mère, à la perception de la mère par rapport à la vaccination, aux distances ménage-centre de santé et ménage-relais communautaire (Tableau I).

**Tableau I:** Opérationnalisation des variables Variables description

Facteurs	Variables	Nature et types des variables	Code	Modalités
**Caractéristiques du ménage**	Niveau de vie	Qualitative ordinale	1	Faible
			2	Moyen
			3	Élevé
			4	Très élevé
	Nombre denfants	Qualitative ordinale	1	1 à 3
			2	4 à 6
			3	7 et plus
**Caractéristiques sociodémo-graphiques des mères**	Groupe d’âge	Qualitative ordinale	1	Moins de 18 ans
			2	18 à 34 ans
			3	35 ans et plus
	Niveau d'instruction	Qualitative ordinale	1	Sans niveau
			2	Alphabétisée
			3	Primaire
			4	Secondaire et Supérieure
	Profession	Qualitative nominale	1	Ménagère
			2	Commerçante
			3	Agent de santé
			4	Artisan/Agricultrice
			5	Fonctionnaire/Enseignante
**Perception des mères**	Perception de la mère sur la complétude vaccinale des enfants	Qualitative nominale	1	Complète
			2	Non complète
			3	Ne sait pas
	Perception sur l'accueil	Qualitative ordinale	1	Très mauvais
			2	Mauvais
			3	Moyen
			4	Bon
			5	Très bon
	Perception sur le délai d'attente	Qualitative ordinale	1	Très court
			2	Court
			3	Moyen
			4	Long
			5	Très long
	Perception sur la distance	Qualitative ordinale	1	Pas du tout longue/ Modérément longue
			2	Longue
			3	Très longue
	Perception sur l'importance de la vaccination	Qualitative ordinale	1	Pas importante du tout/un peu importante
			2	Modérément importante
			3	Importante
			4	Très importante
	Connaissance des maladies du PEV	Qualitative binaire	0 1	Aucune connaissance
				Connaissance
	Lieu de vaccination	Qualitative binaire	1	CS privé
			2	CS public
	Information sur la vaccination	Qualitative binaire	0 1	Non
				Oui
**Caractéristiques sociodémo-graphiques des enfants**	Groupe d’âge (mois)	Qualitative ordinale	1	0 à 2
			2	3 à 5
			3	6 à 8
			4	9 à 15
			5	16 et plus
	Sexe	Qualitative binaire	1	Masculin
			2	Féminin
**Distance**	Distance entre ménages et centres de santé	Qualitative ordinale	1	Pas du tout longue
			2	Modérément longue
			3	Longue
			4	Très longue
	Distance entre ménages et relais communautaires	Qualitative ordinale	1	Pas du tout longue
			2	Modérément longue
			3	Longue
			4	Très longue

CS : centre de santé

## Résultats

Sur les 414 mères enquêtées, 238 possédaient le carnet de vaccination leur enfant soit 57,49 %. Parmi ces 238 mères, 141 étaient à Adjara-Hounvè (59,24 %) et 97 à Ahouicodji (40,76 %). La suite des analyses a porté sur les 238 mères qui possédaient les carnets de vaccination de leur enfant.

### Caractéristiques sociodémographiques de la population d’étude

**Figure 1:**
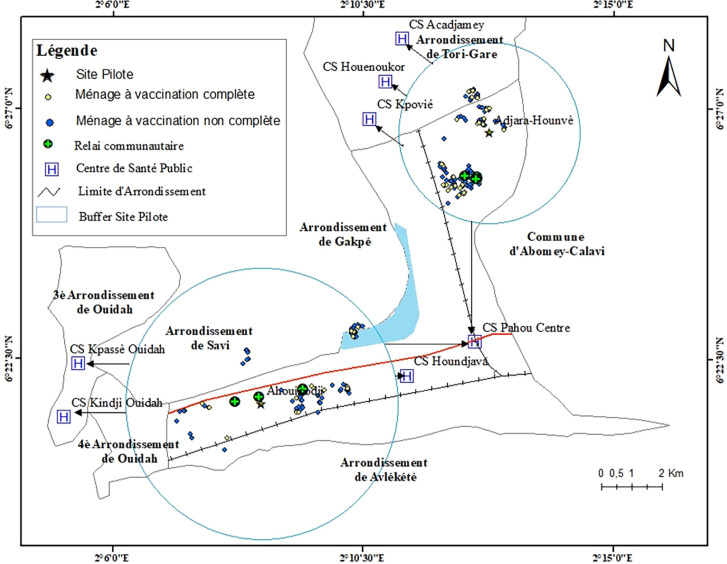
Description du site d’étude, villages d'Adjara-Hounvè et Ahouicodji, Bénin, 2021 Study area mapping, Adjara-Hounvè and Ahouicodji villages, Benin, 2021

La Figure 1 présente la distribution spatiale des ménages ayant des enfants de moins de 5 ans avec ou sans carnet de vaccination, des relais communautaires, et des centres de santé publics que fréquentaient ces ménages pour la vaccination de leurs enfants.

Source : Fond topographique IGN 2018 et enquête de terrain 2021. Réalisation Marlène DAHOUN et Wenceslas VLAVONOU

Une proportion de 39,9 % des ménages enquêtés avait un niveau de richesse moyen. Le nombre moyen d'enfants par ménage était de 3,6 ± 1,7. L’âge moyen des enfants était de 20,6 ± 16,1 mois. La tranche d’âge ayant le plus grand effectif était celle de 16 mois et plus. Le sex-ratio (H/F) des enfants était égal à 1,07. L’âge moyen des mères était égal à 29,1 ± 5,87. Une mère sur quatre enquêtées (37,8 %) avait un niveau d'instruction primaire (Tableau II).

**Tableau II:** Caractéristiques sociodémographiques des ménages, des mères et des enfants enquêtés, villages d'Adjara-Hounvè et Ahouicodji, Bénin, 2021 Socio-demographic characteristics of households, mothers and children, Adjara-Hounvè and Ahouicodji villages, Benin, 2021

Variables	Modalités	Adjara-Hounvè (n = 141)	Ahouicodji (n = 97)	Total (n = 238)
		%	%	%
**Information sur le ménage**				
niveau de vie	Faible	20,6	42,3	29,4
	Moyen	46,1	30,9	39,9
	Élevé	27,0	20,6	24,4
	Très élevé	6,4	6,2	6,3
taille du ménage	1 à 3	58,2	43,3	52,1
	4 à 6	37,6	49,5	42,4
	7 et plus	4,3	7,2	5,5
**Information sur la mère**				
âge (années)	Moins de 18	2,1	2,1	2,1
	18 à 35	76,6	75,3	76,1
	35 et plus	21,3	22,7	21,8
niveau d instruction	Sans niveau	17,7	32,0	23,5
	Alphabétisée	9,9	7,2	8,8
	Primaire	43,3	29,9	37,8
	Secondaire et Supérieure	29,1	30,9	29,8
situation matrimoniale	Célibataire	2,8	11,3	6,3
	Mariée	5,0	3,1	4,2
	Veuve	0,0	6,2	2,5
	Concubine/Union libre	92,2	79,4	87,0
religion	Catholique	28,4	11,3	21,4
	Autres chrétiennes	51,1	37,1	45,4
	Musulmane	3,5	6,2	4,6
	Animiste	7,1	29,9	16,4
	Sans religion	9,9	15,5	12,2
profession	Ménagère	35,5	29,9	33,2
	Commerçante	32,6	44,3	37,4
	Agent de santé	5,0	2,1	3,8
	Agricultrice	1,4	0,0	0,8
	Artisan	18,4	13,4	16,4
	Fonctionnaire	5,7	9,3	7,1
	Enseignante	1,4	1,0	1,3
**Information sur l'enfant**				
âge (mois)	0 à 2	9,9	6,2	8,4
	3 à 5	10,6	16,5	13,0
	6 à 8	7,1	7,2	7,1
	9 à 15	18,4	23,7	20,6
	16 et plus	53,9	46,4	50,8
sexe	Masculin	48,2	56,7	51,7
	Féminin	51,8	43,3	48,3

### Couverture vaccinale complète pour l’âge

Parmi les 238 enfants ayant un carnet de vaccination, 49 avaient une vaccination complète pour leur âge (20,6 %) : 22,0 % à Adjara-Hounvè et 18,6 % à Ahouicodji. Tous les enfants avaient reçu à la naissance le vaccin BCG qui protège contre la tuberculose. Le vaccin contre la poliomyélite et le pentavalent qui protège contre 5 maladies (diphtérie, coqueluche, tétanos, hépatite B et *Haemophilus influenzae* type B), le vaccin contre les infections à pneumocoque (PCV) et le vaccin contre les rotavirus étant à 3 doses au moins, le pourcentage des enfants ayant reçu ces vaccins diminuait au fur et à mesure que le nombre de doses augmentait, soit respectivement pour les 4 doses de la polio 96,64 %, 88,24 %, 78,15 % et 72,27 %, les 3 doses du pentavalent 67,65 %, 62,61 % et 57,14 %, les 3 doses du PCV 14,71 %, 15,13 % et 13,87 % et les 3 doses du vaccin contre le rotavirus 17,65 %, 13,45 % et 12,61 % (Tableau III).

**Tableau III:** Couverture vaccinale par vaccin à Adjara-Hounvè et Ahouicodji, Bénin, 2021, (n = 238) Vaccination coverage in Adjara-Hounvè and Ahouicodji, Benin, 2021

Vaccins	Nombre d'enfants vaccinés	Couverture vaccinale (%)
**BCG**	238	100,00
**Polio 0**	230	96,64
**Poliol /VPO1**	210	88,24
**Polio2 /VPO2**	186	78,15
**Polio3 /VPO3**	172	72,27
**Pental**	161	67,65
**Penta2**	149	62,61
**Penta3**	136	57,14
**PCV13 1**	35	14,71
**PCV13 2**	36	15,13
**PCV13 3**	33	13,87
**Rota1**	42	17,65
**Rota2**	32	13,45
**Rota3**	30	12,61
**VPI**	45	18,91
**RR**	96	40,34
**VAA**	96	40,34

### Raisons de la vaccination non complète pour l’âge

Plusieurs raisons ont été déclarées par les répondants pour justifier l'absence de vaccination complète pour l’âge chez les enfants. Il s'agissait du site de vaccination trop éloigné du lieu de résidence (59,54 %), du manque de moyens financiers (29,78 %) et de l'ignorance de la mère sur la nécessité de la complétude de la vaccination de son enfant (12,76 %).

### Connaissances sur la vaccination et les maladies du PEV

**Figure 2:**
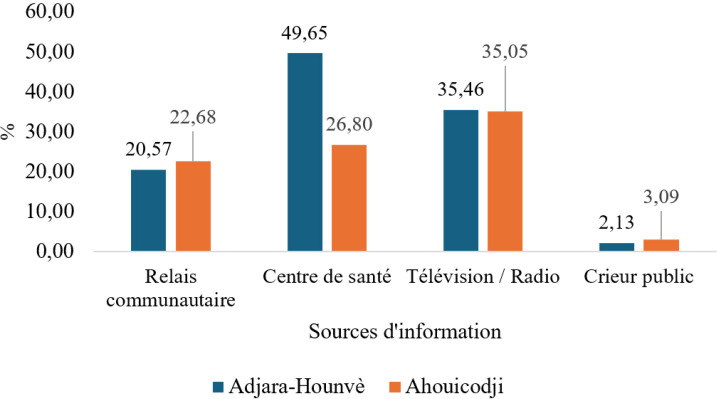
Sources d'information des mères sur la vaccination, villages d'Adjara-Hounvè et Ahouicodji, arrondissement de Pahou, 2021 Mothers’ sources of information on immunization, Adjara-Hounvè and Ahouicodji villages, Pahou district, 2021

Sur les 238 mères enquêtées, plus de trois quarts d'entre elles pensaient que leurs enfants étaient complètement vaccinés. En outre, une proportion de 85,3 % des enquêtées ont reçu au moins une fois des informations sur la vaccination. Selon les données de la Figure 2, les mères recevaient les informations sur la vaccination à Adjara-Hounvè surtout dans les centres de santé (49,65 %) et à Ahouicodji par la télévision ou la radio (35,05 %). Deux mères enquêtées sur trois n'avaient aucune connaissance des maladies cibles du PEV. Les données du Tableau IV montrent que les maladies cibles du PEV les plus connues par les mères étaient l'hépatite B (26,0 %), la rougeole (24,2 %) et la poliomyélite (16,9 %). Les mères faisaient vacciner leurs enfants en majorité dans les centres de santé publics, 95,74 % à Adjara-Hounvè et 100 % à Ahouicodji.

**Tableau IV:** Connaissance des maladies cibles du Programme élargi de vaccination, Adjara-Hounvè et Ahouicodji, arrondissement de Pahou, 2021 Knowledge of diseases targeted by the Expanded Programme on Immunization, Adjara-Hounvè and Ahouicodji villages, Benin, 2021

Variables	Modalités	Adjara-Hounvè (n = 141)	Ahouicodji (n = 97)	Total (n = 238)
		%	%	%
**Connaissance des maladies cibles du PEV**	Aucune connaissance	52,5	76,3	62,2
		Connaissance	47,5	23,7	37,8
**Maladies connues**	Tuberculose	8,5	29,6	13,4
		Poliomyélite (po-lio)	13,6	27,8	16,9
		Rougeole	28,2	11,1	24,2
		Coqueluche	2,3	0,0	1,7
		Tétanos	4,5	3,7	4,3
		Hépatite B	26,6	24,1	26,0
		Fièvre jaune	16,4	3,7	13,4

### Distances entre les ménages et les centres de santé ou les relais communautaires

Les distances minimales et maximales entre les ménages et les relais communautaires de leurs villages étaient de 9,2 m et 2 867,3 m avec une moyenne de 1 256,2 m ± 901,5 m. Par ailleurs, les distances minimales et maximales entre les ménages et les centres vaccinateurs étaient respectivement de 2 003,7 m et 5 447,9 m avec une moyenne de 3 397,3 m ± 736,5 m.

### Perception des mères sur les services de vaccination

L'accueil était perçu comme mauvais ou très mauvais par 17,0 % des mères alors que l'attente était considérée comme longue ou très longue par 65,2 % d'entre elles. Quant à la distance, elle était perçue comme longue ou très longue par 70,9 % des mères (Tableau V).

**Tableau V:** Perception des mères sur la vaccination de leurs enfants, villages d'Adjara-Hounvè et Ahouicodji, Bénin, 2021 Mothers’ perception of their children's vaccination, Adjara-Hounvè and Ahouicodji villages, Benin, 2021

Variables	Modalités	Adjara-Hounvè (n = 141)	Ahouicodji (n = 97)	Total (n = 238)
		%	%	%
**Perception de la mère sur la complétude vaccinale de l'enfant**	Complète	76,6	78,4	77,3
	Non complète	19,9	13,4	17,2
	Ne sait pas	3,5	8,2	5,5
**Perception sur l'accueil**	Très mauvais	5,7	4,1	5,0
	Mauvais	11,3	24,7	16,8
	Moyen	24,1	16,5	21,0
	Bon	47,5	45,4	46,6
	Très bon	11,3	9,3	10,5
**Perception sur l'attente**	Très courte	1,4	3,1	2,1
	Courte	9,9	10,3	10,1
	Moyenne	23,4	47,4	33,2
	Longue	48,9	28,9	40,8
	Très longue	16,3	10,3	13,9
**Perception sur la distance**	Pas du tout longue	0,7	1,0	0,8
	Modérément longue	28,4	30,9	29,4
	Longue	34,0	34,0	34,0
	Très longue	36,9	34,0	35,7
**Importance de la vaccination**	Pas importante du tout	0,0	1,0	0,4
	Un peu importante	3,5	3,1	3,4
	Modérément importante	12,1	29,9	19,3
	Importante	56,7	40,2	50,0
	Très importante	27,7	25,8	26,9

### Facteurs associés à la complétude vaccinale

En analyse univariée, la taille du ménage (p = 0,0059), la profession (p = 0,0035), la perception sur l'importance de la vaccination (p = 0,0360) et la connaissance des maladies du PEV (p < 0,0001) par la mère ainsi que l’âge de l'enfant (p = 0,0002) étaient significativement associés à la complétude vaccinale (Tableau VI).

**Tableau VI:** Facteurs associés à la vaccination complète pour l’âge, analyse univariée (Adjara-Hounvè et Ahouicodji), arrondissement Pahou, 2021 Factors associated with complete vaccination for age, univariate analysis (Adjara-Hounvè and Ahouicodji), Benin, 2021

Variables	Vaccination pour l’âge		Analyse univariée		
	Non complète (n = 189)	Complète (n = 49)	Odds ratio	IC95%	p-value
	%	%			
**Informations liées au ménage**					
**Niveau de vie**					**0,0570**
faible	31,75	20,41		1	
moyen	41,8	32,65	1,22	0,52-2,87	0,6560
élevé	21,16	36,73	2,70	1,13-6,45	0,0250*
très élevé	5,29	10,21	3,00	0,85-10,63	0,0890
**Taille**					0,0059*
1 à 3	47,62	69,39		1	
4 et plus	52,38	30,61	0,40	0,20-0,78	
**Résidence**					**0,5180**
Adjara-Hounvè	58,20	63,26		1	
Ahouicodji	41,80	36,74	0,81	0,42-1,55	
**Informations liées à la mère**					
**Âge (années)**					**0,3680**
moins de 18 ans	1,59	4,08		1	
18 à 34 ans	75,13	79,59	0,41	0,06-2,55	0,3410
35 ans et plus	23,28	16,33	0,27	0,04-1,90	0,1900
**Niveau d'instruction**					**0,0610**
sans niveau	26,99	10,20		1	
alphabétisée	8,99	8,16	2,50	0,58-9,98	0,2280
primaire	35,98	44,90	3,30	1,17-9,31	0,0240*
secondaire et Supérieure	28,04	36,74	3,46	1,20-10,03	0,0220*
**Profession**					0,0035*
ménagère	35,45	24,49		1	
commerçante	38,62	32,65	1,22	0,54-2,77	0,6290
agent de santé	1,06	14,29	19,54	3,62-105,62	0,0010*
artisan/agricultrice	16,93	18,37	1,57	0,60-4,11	0,3580
fonctionnaire/Enseignante	7,94	10,20	1,86	0,57-6,08	0,3040
**Perception sur la couverture vaccinal**e					**0,3900**
complète	72,49	95,92		1	
non complète	21,69	0,00		-	-
ne sait pas	5,82	4,08	0,53	0,11-2,48	0,4200
**Perception sur l'accueil**					**0,7100**
très mauvais	5,83	2,04		1	
mauvais	17,46	14,29	2,33	0,26-21,13	0,451
moyen	21,16	20,41	2,75	0,32-23,87	0,359
bon	44,97	53,06	3,36	0,41-27,30	0,256
très bon	10,58	10,20	2,75	0,28-26,61	0,382
**Perception sur l'attente**					**0,7640**
très courte	1,59	4,08		1	
courte	9,52	12,24	0,50	0,07-3,75	0,5000
moyenne	34,39	28,57	0,32	0,05-2,12	0,2390
longue	40,21	42,86	0,41	0,06-2,65	0,3520
très longue	14,29	12,24	0,33	0,45-2,45	0,2810
**Perception sur la distance entre résidence et centre vaccinateur**					**0,2840**
pas du tout longue/modérément longue	28,57	36,73		1	
longue	33,33	36,73	0,29	0,01-4,79	0,3840
très longue	38,10	26,54	0,18	0,01-3,07	0,2360
**Perception sur l'importance de la vaccination**					0,0360*
très importante	22,75	42,86		1	
importante	51,85	42,86	0,44	0,22-0,89	0,0220*
modérément importante	20,64	14,28	0,37	0,14-0,96	0,0410*
pas importante du tout/un peu importante	4,76	0,00		-	-
**Connaissance des maladies du PEV**					< 0,0001*
non	68,25	38,78		1	
oui	31,75	61,22	3,39	1,77-6,51	
**Information sur l'enfant**									
**Âge (mois)**					0,0002*
0 à 2	4,23	24,49	9,18	3,27-25,73	< 0,0001*
3 à 5	15,34	4,08	0,04	0,09-1,93	0,2660
6 à 8	7,41	6,12		1,31 [0,34-5,05]	0,6940
9 à 15	17,99	30,61	2,70	1,22-5,98	0,0140*
16 et plus	55,03	34,70		1	
**Sexe**					**0,9170**
masculin	51,85	51,02		1	
féminin	48,15	48,98	1,03	0,55-1,94	
**Centre vaccinateur**					**0,0930**
public	98,41	93,88		1	
privé	1,59	6,12	4,04	0,79-20,69	
**Distance ménage - centre de santé**					**0,8810**
pas du tout longue	14,82	16,33		1	
modérément longue	35,45	38,77	0,99	0,38-2,53	0,9870
longue	44,97	38,77	0,78	0,30-0,98	0,6050
très longue	4,76	6,13	1,16	0,25-5,35	0,8430
**Distance ménage - relais communautaire**					**0,4360**
pas du tout longue	40,22	32,65		1	
modérément longue	15,88	22,45	1,74	0,72-4,18	0,2150
longue	21,69	16,33	0,92	0,36-2,34	0,8730
très longue	22,22	28,57	1,58	0,70-3,56	0,2660

* facteurs statistiquement associés à la couverture vaccinale complète pour l’âge

PEV : Programme élargi de vaccination

En analyse multivariée, il ressort que l’âge de l'enfant 0-2 mois et 9-15 mois avait une couverture vaccinale respectivement 8,53 fois et 2,99 fois plus élevée comparée à celle des enfants de 16 mois. De même, le niveau de connaissance des maladies du PEV par la mère a augmenté de 2,20 fois la couverture vaccinale.

Le niveau d'instruction « primaire » (ORa = 3,32; IC95% 1,07-10,25) et la profession de la mère « agent de santé » (ORa = 21,18; IC95% 3,07-145,94) étaient significativement associés à la complétude vaccinale chez l'enfant (Tableau VII).

**Tableau VII:** Facteurs associés à la vaccination complète pour l’âge, analyse multivariée (Adjara-Hounvè et Ahouicodji), arrondissement Pahou, 2021 Factors associated with complete vaccination for age, multivariate analysis (Adjara-Hounvè and Ahouicodji), Benin, 2021

Variables	Vaccination complète pour l’âge		
	Analyse multivariée		
	Odds ratio ajusté	IC95%	p-value
**Niveau d'instruction des mères**			0,0330*
sans niveau		1	
alphabétisée	3,31	0,69-15,76	0,1320
primaire	3,32	1,07-10,25	0,0370*
secondaire et supérieure	2,12	0,63-7,09	0,2191
**Profession des mères**			0,0034*
ménagère		1	
commerçante	1,68	0,64-4,38	0,2850
agent de santé	21,18	3,07-145,94	0,0020*
artisan/agricultrice	1,22	0,41-3,60	0,7100
fonctionnaire/enseignante	2,63	0,63-10,83	0,1800
**Connaissance des maladies du PEV par les mères**			0,0390*
non		1	
oui	2,20	1,03-4,68	
**Âge de l'enfant (mois)**			0,0013*
0 à 2	8,53	2,52-28,85	0,0010*
3 à 5	0,35	0,06-1,82	0,2160
6 à 8	1,66	0,39-6,97	0,4850
9 à 15	2,99	1,24-7,23	0,0150*
16 et plus		1	

* facteurs statistiquement associés à la couverture vaccinale complète pour l’âge

PEV : Programme élargi de vaccination

**Figure 3:**
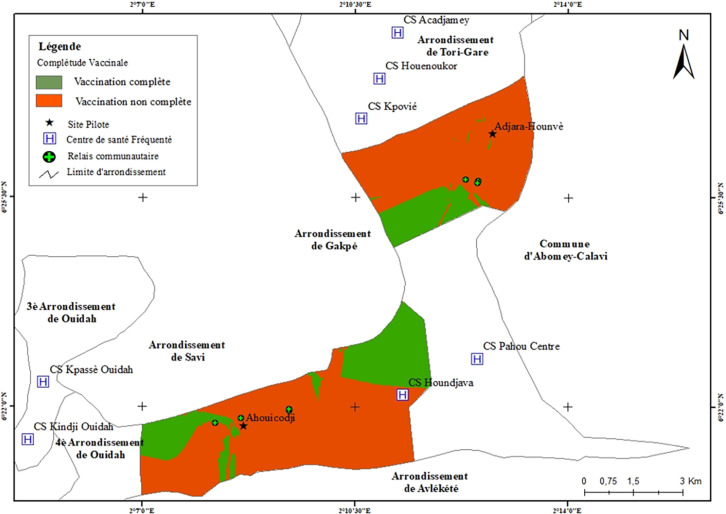
Dynamique spatiale de la vaccination complète pour l’âge chez les enfants de moins de 5 ans dans les villages d'Adjara-Hounvè et Ahouicodji en 2021 Spatial distribution of full immunization for age in children under 5, villages of Adjara-Hounvè and Ahouicodji, Benin, 2021

Les ménages dont les enfants avaient une couverture vaccinale complète étaient regroupés aux extrémités des villages : les deux extrémités pour Ahouicodji et une extrémité pour Adjara-Hounvè et non loin des formations sanitaires. Certains ménages dont les enfants avaient une couverture vaccinale incomplète étaient également situés non loin des formations sanitaires et des relais communautaires (Fig. 3).

**Source : Fond topographique IGN 2018 et enquête de terrain 2021. Réalisation Marlène DAHOUN et Wenceslas VLAVONOU**

## Discussion

Il s'agissait d'une étude exploratoire. En termes de limite, les deux localités choisies ne sont pas représentatives de la zone sanitaire de Ouidah-Kpomassè-Tori Bossito. Il n'est donc pas possible de réaliser une extrapolation des résultats à l'ensemble de cette zone sanitaire. L'absence de carnet de vaccination chez 42,5 % des mères ne permet pas d'apprécier le statut vaccinal de plus du tiers des enfants, ce qui laisse une certaine imprécision de la couverture vaccinale. Cependant, la force de l’étude réside dans le fait qu'elle utilise plusieurs approches méthodologiques descriptive (y compris cartographique) et analytique pour comprendre le faible résultat en termes de couverture vaccinale complète pour l’âge. L'identification des facteurs associés vient renforcer ou compléter les déclarations sur les raisons de l'absence de vaccination complète comme obstacles à l'atteinte des indicateurs de la vaccination. Ces deux approches méthodologiques descriptive et analytique sont complémentaires.

À l'issue de cette étude, il ressort que la couverture vaccinale complète pour l’âge chez les enfants de moins de 5 ans était très faible avec 20,6 %. Cette proportion d'enfants complètement vaccinés était environ 3 fois inférieure à celle enregistrée dans la même région lors de la cinquième édition de l'Enquête démographique de santé (EDS V) (58,9 %) en 2017-2018 et très éloignée du seuil de 80 % fixé par l'OMS dans chaque commune [21]. Une étude portant sur l’évaluation de la qualité de la vaccination chez les enfants de 12 à 24 mois dans la zone sanitaire Adjohoun-Bonou-Dangbo, dans un contexte relativement semblable au sud-ouest du Bénin, en 2021, a révélé des proportions similaires (80,1 % d'enfants de 12-24 mois incomplètement vaccinés) [2].

Une revue précédente, explorant les raisons liées à la non-vaccination et à la sous-vaccination des enfants dans les pays à revenu faible et intermédiaire, a classé les facteurs ou obstacles en plusieurs catégories : les systèmes de vaccination, la communication et l'information, les caractéristiques familiales et les attitudes ou connaissances des parents [32]. Une autre étude les a abordées en termes d'obstacles liés au système de santé, aux parents des enfants et aux soignants [4]. Dans ce travail, les facteurs ou obstacles ont été classés en deux catégories, celle liée au système de santé et celle liée aux parents ou gardiens d'enfants de moins de 5 ans (tuteurs tels que la tante, l'oncle, le grand-père, la grand-mère, autres parents ou amis).

Concernant les obstacles liés au système de santé, le délai d'attente et la distance entre le lieu de résidence et celui de la vaccination étaient considérés comme trop longs. Dans le même sens, une étude menée par Tefera *et al.* en Éthiopie [37] a indiqué que les enfants issus des familles dont le domicile se trouvait à une heure ou plus du site de vaccination étaient moins susceptibles d’être complètement vaccinées (56 %) que les enfants issus des familles dont le domicile se trouvait entre 30 et 59 minutes (67 %). D'autres études au Kenya et au Nigéria [3, 34, 35, 39] ont également révélé que certaines régions d'accès difficile ne disposent pas de structures de santé à proximité pour assurer la vaccination des enfants, ce qui entraînerait la non-réalisation des séries de vaccination recommandées.

Concernant les obstacles liés aux parents ou gardiens d'enfants, le manque de moyens financiers et l'ignorance de la mère sur la nécessité de la complétude de la vaccination de son enfant avaient également été cités. Par ailleurs, le niveau d'instruction, la profession des mères, la connaissance des maladies du PEV par les mères et l’âge des enfants ont influencé statistiquement le statut vaccinal complet pour l’âge. Dans la littérature, la limitation financière est l'un des principaux obstacles à la vaccination des enfants cités par les répondants dans les études réalisées dans plusieurs pays d'Afrique subsaharienne dont le Togo, le Nigéria, le Malawi, l’Éthiopie et la Tanzanie [1,7,9,17,24,26,35,37]. Le manque de moyens financiers est en lien étroit avec le recours aux services de soin et de santé. En effet, dans les pays à faible ou moyen revenu, ce facteur est souvent en lien avec les ignorances de la mère sur les déterminants sociaux de la santé et les mesures de prévention.

De précédentes études ont montré qu'un enfant né d'une mère ayant peu ou pas de connaissances en matière de vaccination ou sur les avantages liés peut ne pas achever la série de vaccins requise [15, 26]. Entre 0 à 2 mois, les différents vaccins administrés aux enfants dont le BCG, le vaccin contre la poliomyélite et le pentavalent se font à la naissance. Les séances d'information, d’éducation et de communication adressées à la mère les premiers jours après la naissance et lors de la consultation post-natale (entre la 6^e^ et la 8^e^ semaine du post-partum) pourraient augmenter la complétude vaccinale à cet âge par rapport aux enfants âgés de 2 à 9 mois. De 9 à 15 mois, les vaccins administrés sont ceux contre la rougeole et la rubéole (RR), et celui contre la fièvre jaune ou anti-amarile (VAA). La rougeole est l'une des maladies les plus connues par la population et la plus contagieuse. Celle-ci étant bien identifiée par un grand nombre de ménages et causant de nombreux décès, les parents recourent à la vaccination contre cette maladie. Ceci explique que la plupart des enfants de 9 à 15 mois ont reçu ce vaccin. La couverture des vaccins nouvellement administrés dans le cadre de la vaccination de routine comme le vaccin contre le méningocoque A (Men A) mérite également d’être évaluée. L'intensification de la communication pour un changement de comportement et la généralisation des stratégies avancées en matière de vaccination pourraient être une solution pour améliorer la couverture vaccinale complète pour l’âge. Concernant le niveau d'instruction, de façon générale, les parents ayant un faible niveau d’éducation montrent plus d'hésitation vis-àvis de la vaccination [1,3,12,21,38]. Au niveau national, selon l'EDS V du Bénin, la couverture vaccinale complète pour l’âge s'améliore avec l’élévation du niveau d'instruction de la mère [21]. Une étude réalisée chez les enfants hospitalisés dans deux hôpitaux de référence pédiatrique à Yaoundé, a montré que le niveau d’étude secondaire augmentait le recours à la vaccination [25] par rapport à des parents dont le niveau scolaire était faible. Dans une étude réalisée dans la région de Kaolack au Sénégal [36], le niveau d'instruction des mères représentait un déterminant social inversement corrélé avec la couverture vaccinale des enfants de 12 à 23 mois (p < 0,05). De façon similaire, le statut vaccinal de l'enfant peut être déterminé par la profession ou le revenu de la mère [10, 36]. Dans une étude sur les déterminants sociaux de la couverture vaccinale de routine, la couverture vaccinale était plus élevée chez les femmes avec activité génératrice de revenus par rapport aux autres. Quant aux femmes sans profession, 84,8 % de leurs enfants ne sont pas complètement vaccinés. Cependant, les études ne sont pas unanimes dans la littérature. Selon d'autres auteurs, les enfants des femmes au foyer auraient une couverture vaccinale plus complète que ceux de femmes d'autres professions telles que les commerçantes ou les employées du secteur public/privé [37]. Les facteurs associés à la vaccination complète pour l’âge chez l'enfant seraient globalement similaires concernant les facteurs liés au système de santé mais fortement dépendants du contexte local concernant les ménages.

## Conclusion

Des résultats de cette étude, il ressort que la complétude vaccinale est très faible chez les enfants de moins de 5 ans. Les facteurs tels que l’âge de l'enfant, un meilleur niveau de connaissance des maladies cibles du PEV par les mères, le niveau élevé d'instruction de la mère et certaines professions de la mère ont augmenté la complétude vaccinale de l'enfant. La distribution spatiale de la complétude vaccinale pour l’âge chez les enfants de 0 à 5 ans a montré une tendance d'homogénéité des comportements de recours à la vaccination. Sur le plan méthodologique (collecte, analyse et interprétation des données), cette étude exploratoire sert de base à la conception du protocole d’évaluation de la couverture vaccinale au niveau national.

## Contribution des auteurs

La conception de l’étude, la rédaction, la révision et validation du protocole ont été faites par DBG, VMW, DM et AB. Les données ont été recueillies par VMW et DM et analysées par DBG, VMW et DM. Le premier draft du manuscrit a été réalisé par DBG. DBG et AB ont apporté leur contribution à la rédaction finale et à la correction du manuscrit. L'article a été validé par tous les auteurs.

## Conflits d'intérêts et principes éthiques

Les auteurs ne déclarent aucun lien d'intérêt. La coordination de la zone sanitaire Ouidah-Kpomassè-Tori Bossito sous le N° 1039ZSOKT/BZ/SA a donné son autorisation à la collecte des données. L'autorisation de recherche du Centre de formation et de recherche en matière de population a été obtenue sous le N° 173-2021/UAC/CEFORP/D/SGE/SA. Les mères ont donné leur consentement éclairé verbal à l’étude. Les données ont été collectées sous anonymat.
